# Phenotypic, Genomic and Functional Characterization Reveals No Differences between CD138^++^ and CD138^low^ Subpopulations in Multiple Myeloma Cell Lines

**DOI:** 10.1371/journal.pone.0092378

**Published:** 2014-03-21

**Authors:** Teresa Paíno, María E. Sarasquete, Bruno Paiva, Patryk Krzeminski, Laura San-Segundo, Luis A. Corchete, Alba Redondo, Mercedes Garayoa, Ramón García-Sanz, Norma C. Gutiérrez, Enrique M. Ocio, Jesús F. San-Miguel

**Affiliations:** 1 Centro de Investigación del Cáncer, Instituto de Biología Molecular y Celular del Cáncer/Consejo Superior de Investigaciones Científicas-Universidad de Salamanca, Salamanca, Spain; 2 Hospital Universitario de Salamanca, Salamanca, Spain; 3 Instituto de Investigación Biomédica de Salamanca (IBSAL), Salamanca, Spain; 4 Clínica Universidad de Navarra; Centro de Investigaciones Médicas Aplicadas (CIMA), Pamplona, Spain; Karolinska Institutet, Sweden

## Abstract

Despite recent advances in the treatment of multiple myeloma (MM), it remains an incurable disease potentially due to the presence of resistant myeloma cancer stem cells (MM-CSC). Although the presence of clonogenic cells in MM was described three decades ago, the phenotype of MM-CSC is still controversial, especially with respect to the expression of syndecan-1 (CD138). Here, we demonstrate the presence of two subpopulations - CD138^++^ (95–99%) and CD138^low^ (1–5%) - in eight MM cell lines. To find out possible stem-cell-like features, we have phenotypically, genomic and functionally characterized the two subpopulations. Our results show that the minor CD138^low^ subpopulation is morphologically identical to the CD138^++^ fraction and does not represent a more immature B-cell compartment (with lack of CD19, CD20 and CD27 expression). Moreover, both subpopulations have similar gene expression and genomic profiles. Importantly, both CD138^++^ and CD138^low^ subpopulations have similar sensitivity to bortezomib, melphalan and doxorubicin. Finally, serial engraftment in CB17-SCID mice shows that CD138^++^ as well as CD138^low^ cells have self-renewal potential and they are phenotypically interconvertible. Overall, our results differ from previously published data in MM cell lines which attribute a B-cell phenotype to MM-CSC. Future characterization of clonal plasma cell subpopulations in MM patients' samples will guarantee the discovery of more reliable markers able to discriminate true clonogenic myeloma cells.

## Introduction

Multiple myeloma (MM) is characterized by the accumulation of malignant plasma cells (PCs) in the bone marrow. Despite recent advances in therapy that contributed to double patients' survival [1], MM remains an incurable disease which may potentially be explained, at least in part, to the persistence of resistant MM cancer stem cells (MM-CSC) with clonogenic potential. The presence of clonogenic cells in MM was described more than 30 years ago [2], but the phenotype of this population is still a matter of debate. It is well known that syndecan-1 (CD138), a heparan sulfate proteoglycan, is expressed by both normal and malignant PCs in most of MM patient samples and cell lines [3], [4], [5], while absent on all earlier B-cells [5], [6], [7], [8]. Interestingly, some authors have described the presence of potential MM-CSC that lacked expression of CD138 both in MM cell lines and patient samples [9], [10], [11]. However, other studies have also demonstrated that CD138^+^ PCs are clonogenic and can engraft in different mice models [12], [13], [14]. It has also been reported that the tumor microenvironment enhances the clonogenicity of human myeloma cells and promotes their de-differentiation towards a more CD138 negative phenotype [15], [16]. Therefore, whether MM-CSCs are CD138^+^ or CD138^−^ is still controversial and multiple factors could be implicated in this particular phenotype. Moreover, it has been recently suggested that the CD138^−^ MM subpopulation seems to represent an apoptotic artifact due to sample handling and procedures [17]. In the present study, we have analyzed eight MM cell lines and we have observed that all of them contain a minor subpopulation of CD138^low^ cells. Overall, our results show that the subpopulation of CD138^low^ cells does not differ from the major CD138^++^ subpopulation regarding phenotypic, genomic and functional features.

## Materials and Methods

### Ethics statement

All animal experiments were conducted according to Institutional Guidelines for the Use of Laboratory Animals of the University of Salamanca (Spain), after acquiring permission from the Bioethics Committee of the University of Salamanca, Spain (Reg. N° 201100030128) and in accordance with current Spanish laws on animal experimentation (RD53/2013).

### Reagents and immunochemicals

Cell-culture media, serum, and penicillin-streptomycin were purchased from Invitrogen (Carlsbad, CA). Bortezomib was provided by Millennium Pharmaceuticals (Cambridge, MA) and melphalan and doxorubicin were obtained from Sigma-Aldrich (St Louis, MO). Annexin-V–FITC was purchased from Immunostep (Salamanca, Spain). May-Grünwald and Giemsa stains were obtained from Merck (Darmstadt, Germany). The origin of the antibodies used in immunocytochemistry and flow cytometry was as follows: anti-CD138-APC (clone B-B4), used for immunocytochemistry and flow cytometry, from Miltenyi Biotec (Auburn, CA); anti-CD20-FITC (clone L27), anti-CD138-PerCP-Cy5 (clone MI15), anti-CD56-APC (clone NCAM16.2), anti-CD45-AmCyan (clone 2D1) and anti-CD38-PE (clone HB7) from BD Biosciences (San Jose, CA, USA); anti-CD19-PacificBlue (clone HIB19) and anti-CD27-PE-Cy7 (clone O323) antibodies from eBioscience (San Diego CA, USA); anti-CD38-AlexaFluor700 antibody (clone HIT2) from Exbio (Vestec, Czech Republic) and anti-CD138-FITC (clone B-A38) from Cytognos S.L. (Salamanca, Spain). The FITC anti-Ki-67 Set was purchased from BD Biosciences (San Diego, CA) and DRAQ5® was obtained from Biostatus (Leicestershire, UK).

### Cell lines, cell culture and morphological characterization

The multiple myeloma cell lines used were: MM1S and MM1R (from Dr. S. T. Rosen, Northwestern University, Chicago, IL) [18]; NCI-H929 (from Dr. J. Teixidó, Centro de Investigaciones Biológicas, Madrid, Spain) [19] and RPMI-8226, U266, RPMI-LR5, U266-LR7 and U266-Dox4 (from Dr. W.S. Dalton, Moffitt Cancer Center, Tampa, FL) [20], [21]. Briefly, cells were cultured in RPMI-1640 medium supplemented with 2 mM L-glutamine, 100 U/mL penicillin, 100 mg/mL streptomycin and 10% fetal bovine serum at 37°C and 5% CO_2_/95% air. Morphological characterization of CD138^++^ and CD138^low^ myeloma cells was performed with May-Grünwald-Giemsa staining.

### Fluorescence-activated cell sorting and flow cytometry

MM cell lines were immunophenotyped on a FACSCanto II cytometer (Becton Dickison Biosciences) using a 7-color immunofluorescence technique [22] with the following combination of monoclonal antibodies (Pacific Blue (PB)/anemonia majano cyan (AmCyan)/fluorescein isothiocyanate (FITC)/peridinin chlorophyllprotein-cyanin 5.5 (PerCP-Cy5.5)/PE-cyanin 7 (PE-Cy7)/allophycocyanin (APC)/alexafluor 700 (AF700)): CD19/CD45/CD20/CD138/CD27/CD56/CD38. Data analysis was performed using the Infinicyt software (Cytognos S.L.). CD138^++^ and CD138^low^ cells were sorted on a FACSAria cytometer (BD Biosciences) after incubation with CD138-APC plus 7AAD. Sorting was performed exclusively on viable cells (7AAD^−^), excluding debris by scatter properties and gating on the lowest and highest 5% CD138-expressing cells. The CD138^++^ and CD138^low^ sorted cells had a final purity >95% (Figure S1). In some experiments the phenotype of the sorted populations was re-analyzed after their expansion *in vitro*. Thus, either CD138^++^ or CD138^low^ RPMI-8226 cells were single-sorted into 96 multiwell plates (1 cell/well) and serially expanded into 48, 24 and 12 multiwell plates for 25 up to 37 days. Afterwards, the phenotype of each clone was analyzed by flow cytometry using CD138-APC plus 7AAD. Apoptosis was determined by double staining with CD38-PE plus CD138-APC followed by incubation with Annexin-V-FITC/7AAD. To analyze the expression of Ki-67 in CD138^++^ and CD138^low^ populations, cells were first surface-stained with CD138-APC. Afterwards, they were fixed and permeabilized with the Cytofix/Cytoperm™ Fixation/Permeabilization Solution Kit (BD Biosciences) and then stained either with Ki-67-FITC or isotype control. To analyze the cell cycle, cells were first incubated with CD138-FITC followed by DRAQ5® and measured on a FACSCanto II cytometer. The expression of aldehyde dehydrogenase (ALDH) was assessed using the Aldefluor Kit (StemCell Technologies, Grenoble, France) following the manufacturer's instructions with further staining with CD138-PerCP-Cy5.

### Immunocytochemistry

After FACS sorting, cells were collected, washed with PBS, centrifuged at 800 g for 3 minutes and then resuspended in 200 μl of PBS. An aliquot was taken from each sample and placed on glass slides coated with poly-L-lysine followed by 15 minutes incubation in a cell culture incubator. Then, cells were fixed with 4% paraformaldehyde for 30 minutes, blocked with BSA 100 μg/ml for 1 h at room temperature and incubated with anti-CD138-APC (1∶100) overnight at 4°C. Glass slides were then mounted using VECTASHIELD Mounting Medium (Vector Laboratories, Burlingame, CA). Finally, images were collected under Zeiss confocal microscope equipped with 63×/1.4 Oil Plan-APOCRHOMAT DIC.

### Real time quantitative PCR (qRT-PCR)

Total RNA was extracted from CD138^++^ and CD138^low^ myeloma cells using an RNeasy Mini Kit (Qiagen, Valencia, USA) following the manufacturer's protocol. RNA quality and quantity were assessed with the RNA Nano LabChip (Agilent Tech. Inc., Palo Alto, CA, USA). The retrotranscription reaction was performed using SuperScript™ III First-Strand Synthesis SuperMix (Invitrogen) according to the manufacturer's recommendations. Finally, qRT-PCR was performed to detect the target gene (*SDC1*) using iQ™ SYBR GreenSupermix kit (BioRad). SYBR green qRT-PCR was performed using Bio-Rad iQ5 PCR detection system with the following gene-specific primers: *GAPDH*, forward 5′-CAGGGCTGCTTTTAACTCTGGTAA-3′ and reverse 5′-GGGTGGAATCATATTGGAACATGTA-3′; *SDC1*, forward 5′-GGCTGTAGTCCTGCCAGAAG-3′ and reverse 5′-GTTGAGGCCTGATGAGTGGT-3′. Relative gene expression was calculated by the 2^−ΔCt^ method, ΔCt = Ct (gene)−Ct (GAPDH).

### Cell viability assay

Viability of multiple myeloma cells was assessed using 3-(4,5-dimethylthiazol-2-yl)-2,5-diphenyltetrazolium bromide (MTT) colorimetric assay as previously described [23]. Briefly, CD138^++^ or CD138^low^ RPMI-8226 sorted cells were plated into 96-well culture dishes (20000 cells/well) in the absence (control) or presence of 10 nM bortezomib, 10 μM melphalan or 250 nM doxorubicin and incubated for 24 and 48 hours.

### Microarray RNA analyses

RNA was isolated from CD138^++^ (n = 3) and CD138^low^ (n = 3) RPMI-8226 cells using an RNeasy Mini Kit (Qiagen, Valencia, USA) following the manufacturer's protocol. RNA was hybridized to Human Gene 1.0 ST array (Affymetrix) according to Affymetrix protocols [24]. Microarray data were normalized using the Robust Multichip Analysis (RMA) algorithm implemented in the Affymetrix Expression Console. Data analysis was carried out using DNA-Chip Analyzer software (DChip). The comparison criteria used in DChip analysis were fold change E/B≥2 or B/E≥2, mean difference E-B>100 or B-E>100 and the lower 90% confidence bound of fold-change was used. All microarray data have been deposited with the Gene Expression Omnibus (http://www.ncbi.nlm.nih.gov/geo/; accession number GSE49482).

### Identification of IGH rearrangements

Complete VDJH rearrangements were amplified using two multiplexed tubes containing VH-family specific primers for FR1 and FR3 regions with a JH consensus primer according to the BIOMED-2 Concerted Action [25]. All reactions were carried out in 25 μL containing 100 ng of DNA samples and 10 pm of each primer. Then the clonal nature of the PCR fragments was confirmed by Gene-Scanning analysis in an ABI 3130 DNA Sequencer (Applied Biosystems, Foster City, CA, USA).

### Copy number abnormalities (CNA) studies

DNA was isolated from CD138^++^ (n = 3) and CD138^low^ (n = 3) RPMI-8226 cells using GenElute Mammalian Genomic DNA Miniprep Kit (Sigma-Aldrich) following the manufacturer's protocol. The detection of CNA was investigated using the CytoScan HD array (Affymetrix, Santa Clara, CA, USA) which includes more than 2.6 million copy number markers and 750.000 SNPs. Briefly, genomic DNA was digested with Nsp I restriction endonuclease, ligated to adaptors that recognize the cohesive four base pair overhangs and amplified by PCR. PCR product was purified and fragmented with DNAse I and then end-labeled using terminal deoxynucleotidyl transferase and hybridized to the CytoScan HD array. The arrays were processed using the Fluidics Station 450, GeneChip Scanner 3000 7 G and AGCC (AffymetrixGeneChip Command Console Software).

For the analysis we employed the Affymetrix Chromosome Analysis Suite (ChAS) Software v2.0 and Nexus Copy Number Software Version 7.0 (BioDiscovery, El Segundo, CA, USA). The complete data set was analyzed by visual inspection using ChAS software. CNA were reported when the three following criteria were achieved: minimum of 50 markers per segment, 200 Kb minimum genomic sizes and 50% overlap with known copy number variants (Database of Genomic Variants). Nexus software was employed to obtain the presented Figures. All microarray data have been deposited with the Gene Expression Omnibus (accession number GSE49483).

### Myeloma xenografts

For serial engrafting assays, either sorted CD138^++^ or CD138^low^ RPMI-8266 cells were subcutaneously inoculated into the right flank of 6–7 week old female CB17-SCID mice (Charles River, Spain) as previously described [26] to generate primary tumors (5 mice were inoculated per group with 3×10^4^ cells) and subsequently cells isolated from primary tumors were serially transplanted into new mice to generate secondary tumors (6 mice were inoculated per group with 3×10^6^ cells). Caliper measurement of the tumor diameters were performed three times per week and the tumor volume was estimated as the volume of an ellipse using the following formula: V = 4/3 π × (a/2) × (b/2)^2^, where “a” and “b” correspond to the longest and shortest diameter, respectively. Animals were sacrificed when their tumor diameter was ≤2 cm. To investigate the phenotype of the generated tumors, we mechanically minced and filtered the tumors through 40 μm cell-strainers and stained the obtained cell suspension with CD38-FITC plus CD138-APC. 7AAD was used also to exclude dead cells.

### Statistical analysis

Statistical analysis was performed using the SPSS-20.0 software (SPSS, Chicago, IL, USA). Significant differences between groups were assessed by Mann-Whitney U tests.

## Results

### CD138 expression in MM cell lines

**Figure 1:**
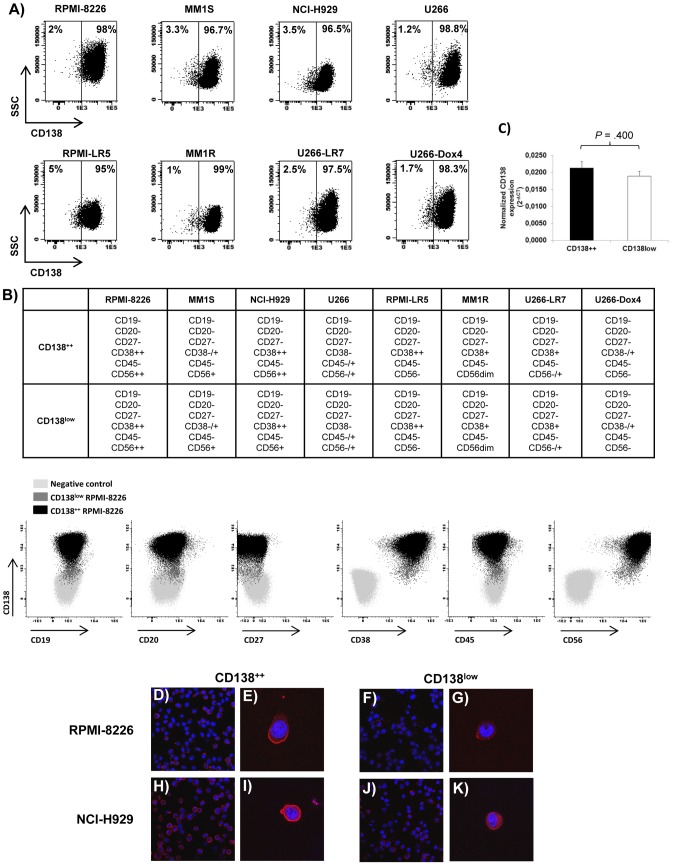
CD138 expression in MM cell lines. (A) Dot plots showing the percentage of CD138^++^ and CD138^low^ cells in MM cell lines. (B) Top: summary of the expression of CD19, CD20, CD27, CD38, CD45 and CD56 in CD138^++^ and CD138^low^ subpopulations in MM cell lines. Code: – (negative); −/+ (heterogeneity); dim (weak positive); + (positive); ++ (high positive). Bottom: representative dot plots corresponding to non-stained RPMI-8226 cells (negative control; light grey) and stained CD138^++^ (black) and CD138^low^ (dark grey) RPMI-8226 cells. (C) Expression of CD138 by real-time quantitative PCR in CD138^++^ and CD138^low^ RPMI-8226 subpopulations. Relative values were calculated by the 2^−ΔCt^ method (ΔCt = Ct_(Gene)_−Ct_(GAPDH)_). The *GAPDH* gene was used as a control gene. Results are expressed as the means ± SEM (n = 3). (D–K) Confocal images corresponding to the immunocytochemistry for CD138 (red) in CD138^++^ and CD138^low^ RPMI-8226 and NCI-H929 cells. Nuclear DNA was stained with DAPI (blue). Magnification of the lens, 63x. Specific “4× zoom” was made in E, G, I, K.

We analyzed the phenotypic profile of eight MM cell lines (RPMI-8226, MM1S, NCI-H929, U266, RPMI-LR5, MM1R, U266-LR7 and U266-Dox4) to investigate the presence of CD138^−^ or CD138^low^ MM cells. All eight MM cell lines contained two populations with different intensity of CD138 expression: one with bright staining (CD138^++^) that represented around 95–99% of all MM cells and a minor fraction showing dim intensity (CD138^low^) that represented 1–5% (Figure 1A). No differences were observed between these two populations regarding other myeloma-related markers (CD38, CD45, CD56) and both subpopulations were negative for the B-cell markers CD19, CD20 and CD27 (Figure 1B). Moreover, in one cell line (RPMI-8226) we analyzed the morphology of both subpopulations and CD138^low^ cells did not display different morphological characteristics as compared to CD138^++^ cells (Figure S2). The expression of CD138 was also explored by alternative techniques in two cell lines (RPMI-8226 and NCI-H929). By qRT-PCR, no differences were noted in CD138 mRNA levels between CD138^++^ and CD138^low^ RPMI-8226 cells (Figure 1C). However, by immunocytochemistry a clear higher surface staining for CD138 in the CD138^++^ subpopulation from both RPMI-8226 and NCI-H929 cells was found (Figures 1D–K) in accordance with the results observed by flow cytometry. It should be noted that neither CD138^++^ nor CD138^low^ subpopulations contained apoptotic nuclei (Figures 1D–K) and in fact both were negative for annexin-V and 7AAD (Figure S3).

### Genomic characterization of CD138^++^ and CD138^low^ subpopulations

**Figure 2:**
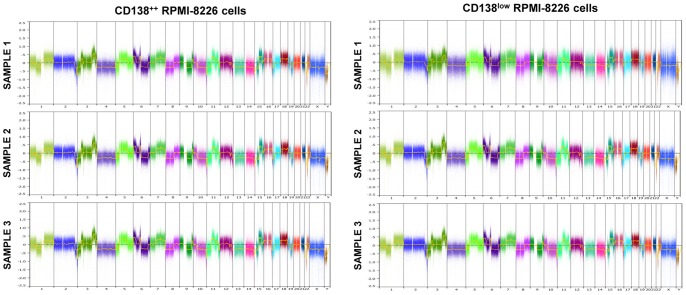
Copy number abnormalities analysis of CD138^++^ and CD138^low^ cells. Log ratio plot of all chromosomes corresponding to CD138^++^ RPMI-8266 cells (n = 3; left panel) and CD138^low^ RPMI-8266 cells (n = 3; right panel) on the basis of Cytoscan HD array generated with Nexus.

To confirm the clonal relationship among CD138^++^ and CD138^low^ subpopulations, we analyzed the *IGH* rearrangements of CD138^++^ RPMI-8226 cells (n = 2) and CD138^low^ RPMI-8226 cells (n = 2). The monoclonal VDJH rearrangement was present in all samples with the same pattern and size in the GeneScan analysis (340 bp for FR1 and 131 bp for FR3), demonstrating a common clonal origin of CD138^++^ and CD138^low^ subpopulations. To explore if phenotypically distinct CD138^++^ and CD138^low^ subpopulations had different genomic changes, we comparatively studied CNA of CD138^++^ RPMI-8226 (n = 3) and CD138^low^ RPMI-8226 (n = 3) subpopulations, but no relevant differences were observed (Figure 2). Finally, we compared the gene expression profile of CD138^++^ and CD138^low^ RPMI-8226 cells. As shown in Table 1, only three genes (*HLA-DRA*, SNORD14E and *CXCL10*) were differentially expressed between the two populations, and specifically overexpressed in the CD138^low^ cells.

**Table 1:** Genes differentially expressed in CD138^low^ RPMI-8226 *versus* CD138^++^ RPMI-8226 cells.

Probe set	Fold change	Gene Title	Gene Symbol
8118548	2,25	major histocompatibility complex, class II, DR alpha	HLA-DRA
7952335	2,29	small nucleolar RNA, C/D box 14E	SNORD14E
8179481	2,37	major histocompatibility complex, class II, DR alpha	HLA-DRA
8178193	2,96	major histocompatibility complex, class II, DR alpha	HLA-DRA
8101126	4,14	chemokine (C-X-C motif) ligand 10	CXCL10

### Proliferation of CD138^++^ and CD138^low^ subpopulations

**Figure 3:**
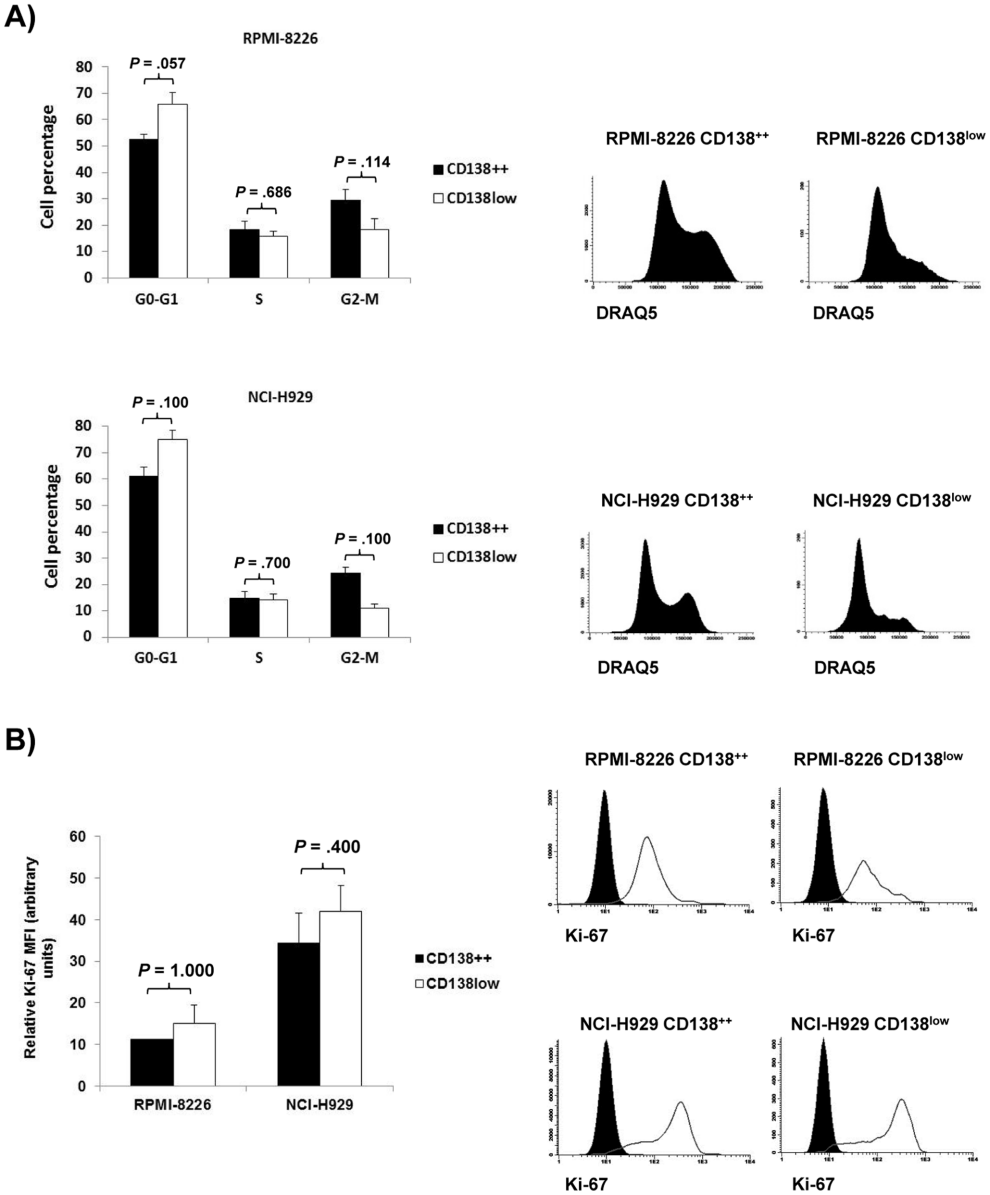
Proliferation of CD138^++^ and CD138^low^ subpopulations. (A) Left: Cell percentage of CD138^++^ and CD138^low^ RPMI-8226 or NCI-H929 cells in G0-G1, S and G2-M phases. The results are expressed as the means ± SEM of at least three independent experiments. Right: Representative DRAQ5 histograms for each indicated population. (B) Left: Relative Ki-67 MFI of CD138^++^ and CD138^low^ RPMI-8226 and NCI-H929 cells with respect to isotype control. Results are expressed as the means ± SEM of three independent experiments. Right: Representative Ki-67 histograms for each indicated population. Filled histograms (isotype control); open histograms (Ki-67).

We analyzed the cell cycle of CD138^++^ and CD138^low^ subpopulations in two cell lines (RPMI-8226 and NCI-H929) and we observed that none of them is truly quiescent, although the CD138^low^ subpopulation shows a numerically -not significantly- lower percentage of cells in G2-M phase as compared to the CD138^++^ subpopulation (Figure 3A). These results were further confirmed by Ki-67 staining. Both CD138^++^ and CD138^low^ subpopulations in RPMI-8226 and NCI-H929 cell lines showed considerable and similar levels of Ki-67 (Figure 3B).

### Study of ALDH expression and drug sensitivity in CD138^++^ and CD138^low^ subpopulations

**Figure 4:**
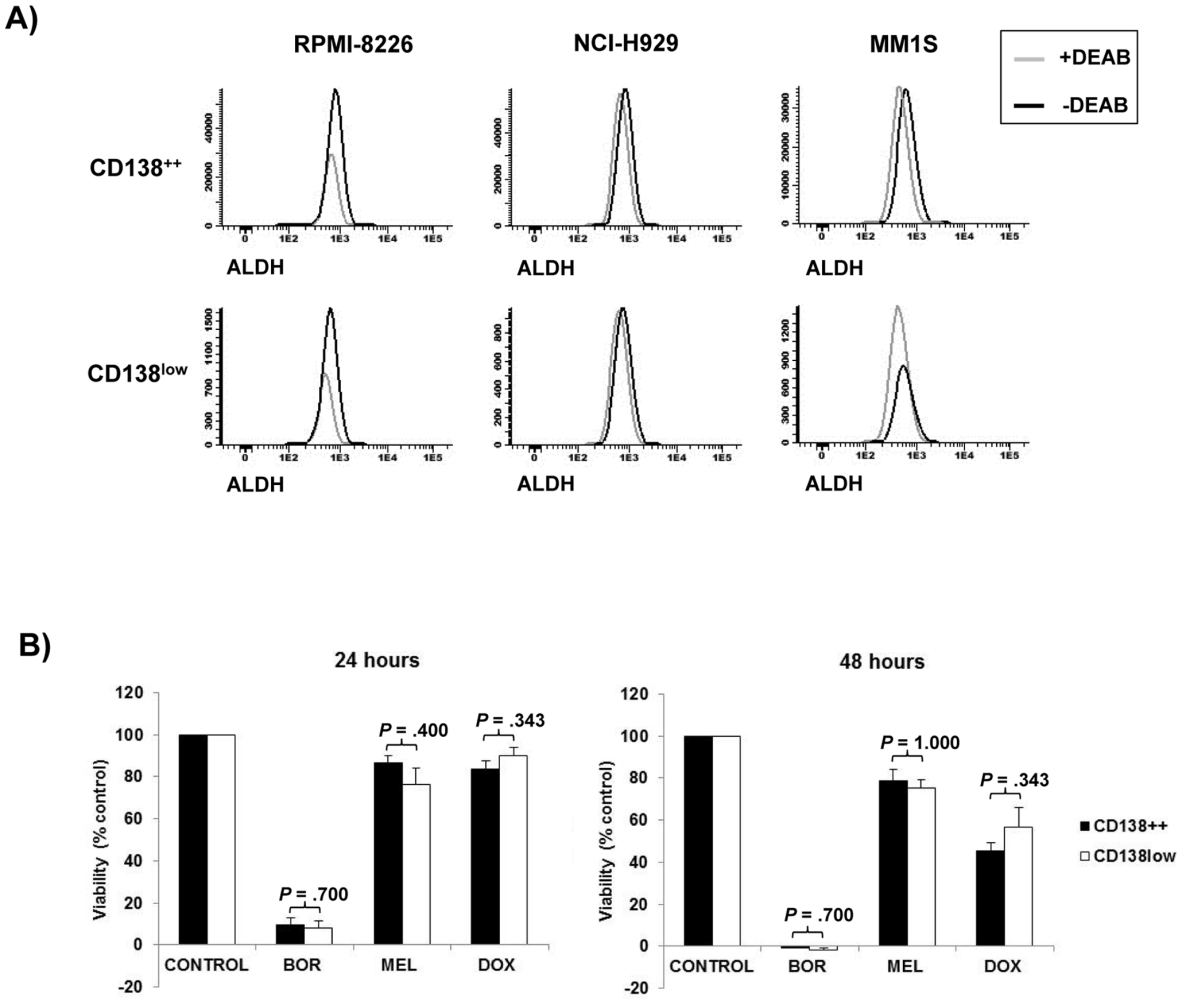
Study of aldehyde dehydrogenase expression and drug sensitivity in CD138^++^ and CD138^low^ subpopulations. (A) Single parameter histograms illustrating the expression of ALDH in the presence or absence of the ALDH inhibitor DEAB (diethylaminobenzaldehyde) in CD138^++^ and CD138^low^ subpopulations of the cell lines RPMI-8226, NCI-H929 and MM1S (B) Sorted CD138^++^ or CD138^low^ RPMI-8226 cells were incubated in the absence (control) or presence of bortezomib (10 nM), melphalan (10 μM) or doxorubicin (250 nM) for 24 and 48 hours. After the incubation time, cell viability was measured by MTT assay and the percentage of cell viability was calculated considering control as 100%. Results are the means ± SEM of at least three independent experiments.

Since drug resistance is a hallmark of stem cells [27], [28] we next studied the expression of the drug resistance and stem cell-associated marker ALDH [29] in CD138^++^ and CD138^low^ cells. In three different cell lines (RPMI-8226, NCI-H929 and MM1S) the expression of ALDH was barely noted in both CD138^++^ and CD138^low^ subpopulations with no differences between them (Figure 4A). To compare the sensitivity of CD138^++^ and CD138^low^ cells to different anti-myeloma drugs we sorted both subpopulations from the RPMI-8226 cell line and incubated the two subpopulations in the absence (control) or presence of bortezomib, melphalan or doxorubicin. No differences in the sensitivity of CD138^++^ and CD138^low^ cells to any of the tested drugs was found (Figure 4B).

### Tumorigenic potential of CD138^++^ and CD138^low^ subpopulations

**Figure 5:**
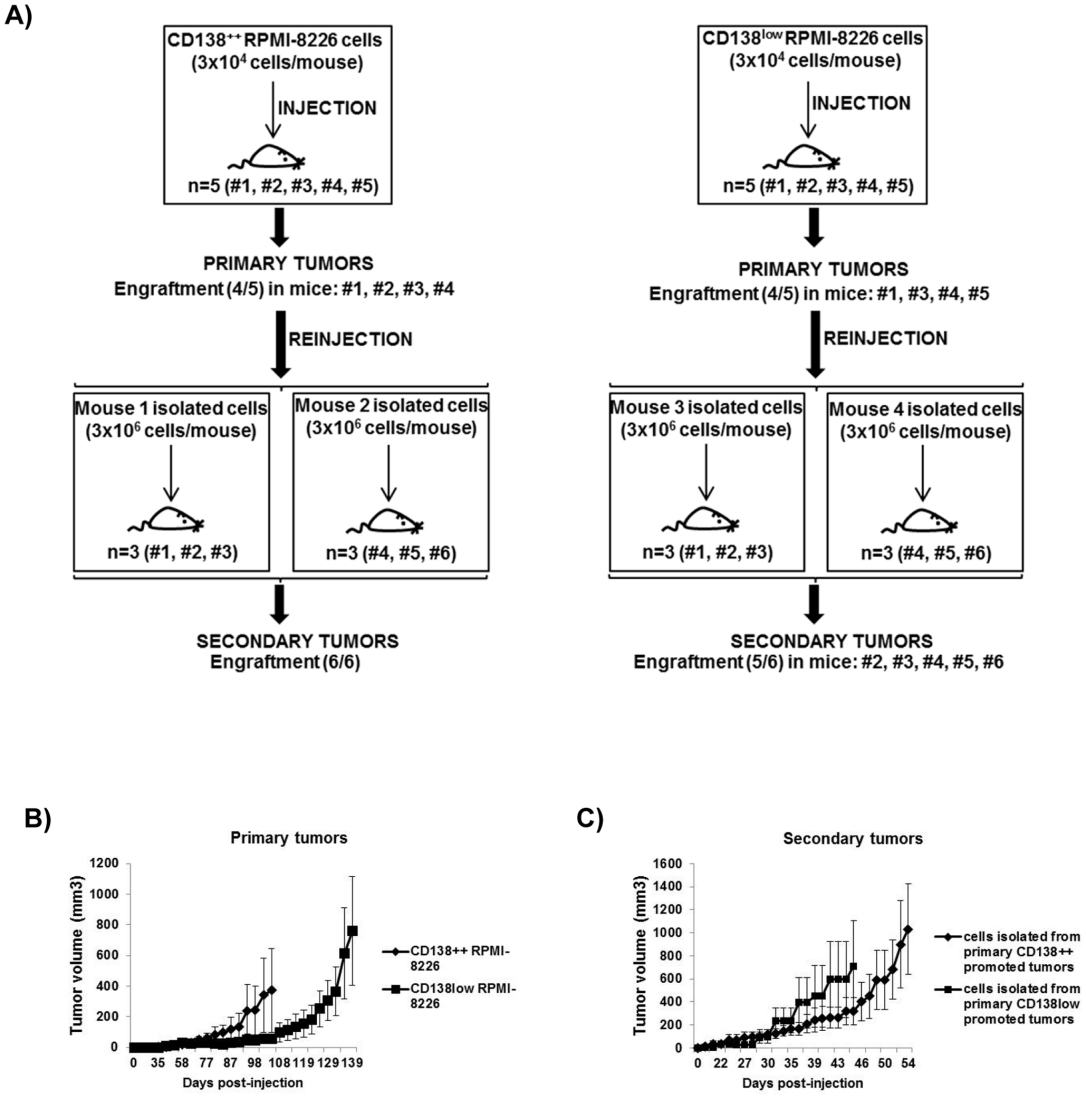
Tumorigenic potential of CD138^++^ and CD138^low^ subpopulations. (A) 3×10^4^ sorted CD138^++^ RPMI-8226 or CD138^low^ RPMI-8226 cells were subcutaneously injected into CB17-SCID mice to generate primary tumors. Subsequently, 3×10^6^ cells isolated from selected primary tumors were serially transplanted into new CB17-SCID mice to generate secondary tumors. The engraftment efficacy is indicated in each case. (B, C) Tumor growth curves for CB17-SCID mice that developed measurable primary and secondary tumors. Growth curves represent tumor volumes (means ± SEM; n = 4–6) until the time point in which the first mouse in every group is sacrificed.

We compared the engrafting potential of CD138^++^ and CD138^low^ RPMI-8226 cells in a serial transplantation assay in CB17-SCID mice. Initially, the same number (3×10^4^) of sorted CD138^++^ and CD138^low^ cells were subcutaneously injected into CB17-SCID mice to generate primary tumors, and subsequently the cells isolated from primary tumors were serially transplanted into new mice (3×10^6^ cells/mouse) to generate secondary tumors (Figure 5A). No significant differences were found for primary and secondary tumors neither in the growth curves (Figures 5B–C) nor in the engrafting capacity (Figure 5A).

### CD138^++^ cells give rise to CD138^low^ cells and vice versa

**Figure 6:**
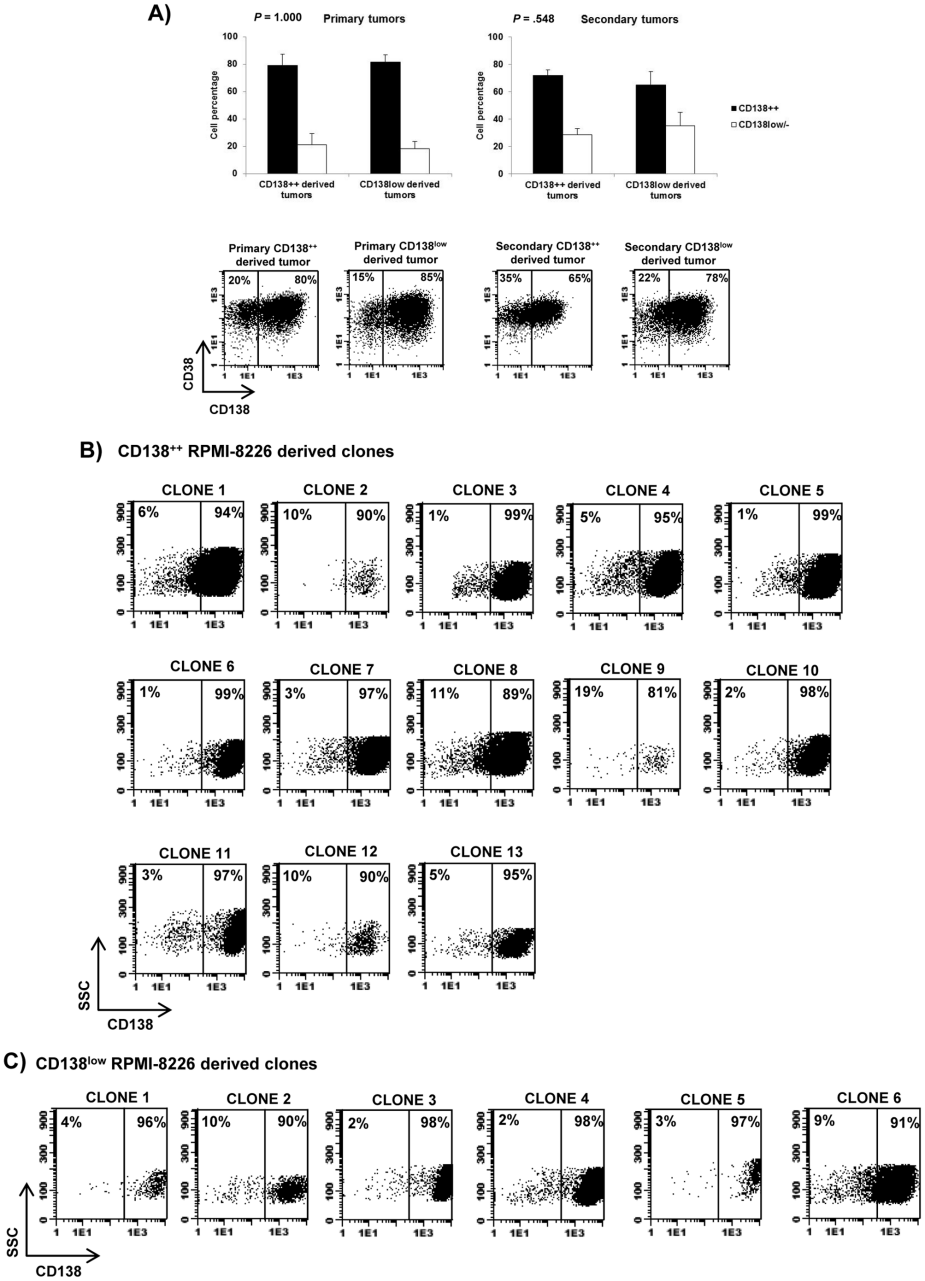
CD138^++^ cells give rise to CD138^low^ cells and *vice versa*. (A) The graphs represent the percentage of CD38^++^CD138^++^ and CD38^++^CD138^low/-^ cells of primary and secondary CD138^++^ RPMI-8226 and CD138^low^ RPMI-8226 derived tumors. Results are expressed as the means ± SEM (n = 4-6). A representative dot plot for every group of tumors is shown and the percentage of CD38^++^CD138^++^ and CD38^++^CD138^low/-^ is indicated in each case (B, C) Phenotype of each individual clone derived from CD138^++^ RPMI-8226 or CD138^low^ RPMI-8226 cells. The percentage of CD138^++^ and CD138^low^ cells is indicated in each dot plot.

Since differentiation potential is a characteristic of stem cells [30], we wanted to know if CD138^++^ cells give rise to CD138^low^ cells or *vice versa*. To this aim, we analyzed the phenotype of the tumors derived from purified CD138^++^ RPMI-8226 or CD138^low^ RPMI-8226 cells. As shown in Figure 6A, primary and secondary tumors derived from both populations contained similar percentages of CD138^++^ and CD138^low/-^ cells, indicating that, *in vivo*, each population is able to give rise to the other. To confirm these results and to dismiss that they are due to contamination of the sorted populations injected into the mice, we performed an *in vitro* experiment of single-cell sorting. Thus, we single-sorted either CD138^++^ or CD138^low^ RPMI-8226 cells into 96 multiwell plates (1 cell/well) and allowed them to expand for a period of time between 25–37 days. We found a higher number of clones derived from CD138^++^ subpopulation (13 clones out of 96) as compared to CD138^low^ subpopulation (6 clones out of 96). The phenotypic analysis of all clones showed that, independently of the initiating cell (CD138^++^ or CD138^low^), every clone contained CD138^++^ and CD138^low^ cells (Figures 6B and 6C). Overall, these results indicate that CD138^++^ cells give rise to CD138^low^ cells and *vice versa*.

## Discussion

Although the presence of clonogenic cells in MM was described more than 30 years ago [2] the phenotype of the resistant putative MM-CSC is still a matter of debate. In this work, we have focused on myeloma cells with low expression of the maturation associated surface marker CD138. Our results show that the minor subpopulation of CD138^low^ cells contained in MM cell lines does not differ from the major CD138^++^ subpopulation regarding phenotypic, genomic and functional features.

An immature phenotype is a typical hallmark of cancer stem cells [31]. However, in MM there is still an ongoing controversy with respect to the phenotype of MM-CSC. Thus, some authors have suggested that MM-CSC are CD138 negative [9], [10], [11], whereas others have demonstrated the existence of CD138 positive plasma cells with stem cell properties [12], [13], [14], [32]. Traditionally, a better understanding of cancer stem cell properties has been possible, in part, by analyzing cell lines obtained from different types of tumors [33], [34], [35]. Here, we investigated in a panel of eight MM cell lines for the presence of a CD138^low/-^ population and we analyzed its biological characteristics. As previously suggested by other authors [9], [16], [36], [37] we confirmed that all cell lines contain a minor subpopulation of CD138^low^ cells. However, it has been recently described that infraexpression of CD138 in MM cells may be an artifact due to apoptotic induction [17]. Our results show that the CD138^low^ subpopulation is viable as demonstrated by DAPI-nuclear as well as Annexin-V/7ADD staining.

The presence of CD138^low/-^ cells in myeloma is intriguing because it could imply a less differentiated state. In fact, it has been previously reported that the interaction of myeloma cells with different types of cells of the bone marrow microenvironment promotes a reduction of CD138 expression, a less differentiated morphology and an increase of the B-cell associated transcription factor, Bcl6 [15], [16], [38]. Here, we have detected the presence of myeloma cells with low expression of CD138 in the absence of bone marrow microenvironment cells and under these circumstances, the population of CD138^low^ cells does not seem to represent a more immature compartment since it does not express the B-cell associated markers CD19, CD20 and CD27 and it does not show a less differentiated morphology. Moreover, VDJH rearrangement analysis, CNA analysis and gene expression profiling did not show relevant differences between CD138^++^ and CD138^low^ cells. In fact, the absence of substantial differences in the gene expression profiling of both populations has also been revealed using single molecule microarray in a recent study [39]. Furthermore, the higher or lower surface expression of CD138 in MM cells seems to be regulated at the protein rather than transcriptional level, as demonstrated by similar CD138 mRNA levels found in the two subpopulations. It should be noted that our *in vitro* and *in vivo* results demonstrate the bidirectional origin of the two populations since CD138^++^ cells give rise to CD138^low^ cells and *vice versa*. These data suggest a dynamic between the two phenotypic subpopulations that tend to equilibrium, and could be responsible of the heterogeneity typically found in MM cells, as previously demonstrated through statistical modeling in breast cancer cell lines [33]. In fact, it has been demonstrated in myeloma patient samples the presence of interconvertible phenotypic and functional states responsible of myeloma-propagating activity and drug resistance [40].

Apart from the immature phenotype, other hallmarks of normal and cancer stem cells are quiescence, chemoresistance and potential to self-renew [41], [42]. Here, we have compared the cell cycle of CD138^++^ and CD138^low^ cells and found that none of them is quiescent as demonstrated by a considerable percentage of cells (≥25%) in the proliferative phases (S and G2-M) of the cell cycle. This was further confirmed by substantial expression of the proliferation-associated marker Ki-67. Nevertheless, we observed a numerically -not significantly- lower percentage of cells in G2-M phase in CD138^low^ cells as compared to the CD138^++^ fraction that could be the cause of the lower number of clones expanded *in vitro* from the CD138^low^ subpopulation. These results are in line with previous data by Fuhler and col. in RPMI-8226 cells [16], but differ from those by Matsui and col. who found that the CD138 negative subpopulation within RPMI-8226 and NCI-H929 cell lines is mainly quiescent (>98% cells in G0–G1) [10], but paradoxically express higher levels of Ki-67 than CD138 positive cells [9]. The proliferation rate of CD138^+^ and CD138^−^ myeloma cells has also been described in primary patient samples with discordant results. Reid and col. observed a higher proliferation rate of CD38^++^CD138^−^ myeloma cells as compared with CD38^++^CD138^+^ myeloma cells [43], whereas Chaidos and col. identified a so-called pre-plasma cell compartment (CD38^++^CD138^−^) that is relatively quiescent [40].

The chemoresistance of cancer stem cells is mainly associated to their specific properties such as quiescence [28], expression of multidrug resistance efflux pumps [44] and expression of detoxifying enzymes [45]. Here, we investigated the expression of the detoxifying enzyme ALDH and, in contrast to previous observations [10], we have not found higher ALDH expression in CD138^low^ cells as compared to CD138^++^ cells. Moreover, CD138^++^ and CD138^low^ RPMI-8226 cells displayed similar sensitivity to different antimyeloma drugs. Overall, these results indicate that the CD138^low^ subpopulation within myeloma cell lines does not represent a specific chemoresistant compartment. In fact, our results support recent data by Jakubikova and col. who found a lack of correlation between the drug resistant “side population” and CD138^-/low^ subpopulations in MM cell lines [37].

As previously mentioned, the potential to self-renew is another hallmark of stem cells [41], [42]. This specific property is linked to the potential of stem cells to serially engraft into immunocompromised mice [46]. In MM, the first evidence of self-renewal pointed to the capacity of circulating cells to engraft into NOD-SCID mice [47]. Nevertheless, later studies have been controversial, with some authors demonstrating the tumorigenic potential of post-germinal center memory B-cells [9], [10] while others of MM plasma cells [12], [14], [32], [40], [48], [49], [50]. Here, we demonstrated in a serial transplantation assay in SCID mice that both CD138^++^ and CD138^low^ RPMI-8226 cells have tumorigenic and self-renewal potential. These results are in line with previous studies in which the tumorigenic potential of CD19^−^CD38^+^CD138^+^ and CD19^−^CD38^+^CD138^−^ primary plasma cells was compared and both populations were able to engraft into immunocompromised mice [32], [40].

In conclusion, our results show that the minor CD138^low^ subpopulation in MM cell lines does not represent an immature B-cell compartment and has similar genomic and functional profile as compared to the major CD138^++^ fraction. Both CD138^++^ and CD138^low^ subpopulations have self-renewal potential, similar drug sensitivity and are phenotypically interconvertible. Therefore, these results highlight the need to explore new and more reliable markers able to discriminate true clonogenic myeloma cells.

## Supporting Information

Figure S1 **Gating strategy for sorting CD138^++^ and CD138^low^ subpopulations in RPMI-8226 cells.** Top panel: viable cells were gated as 7AAD^-ve^ (R1) and subsequently debris were eliminated by scatter properties (R2). Finally, the lowest and highest 5% CD138-expressing cells were gated on R2. Bottom panel: CD138^++^ and CD138^low^ sorted cells had a final purity >95% in the post-sort analysis.
(DOCX)

Figure S2 **Morphology of CD138^++^ and CD138^low^ RPMI-8226 cells.** May-Grünwald-Giemsa staining of sorted CD138^++^ and CD138^low^ RPMI-8226 cells. Scale bar  = 10 μm.
(DOCX)

Figure S3 **Analysis of apoptosis in CD138^++^ and CD138^low^ subpopulations in RPMI-8226, NCI-H929, MM1S and U266 cell lines.** Non-apoptotic cells were gated as annexin-V^-ve^/7AAD^-ve^ (R1) and subsequently debris were eliminated by scatter properties (R2). The third dot plot for every MM cell line corresponding to R2 shows the percentage of CD138^low^ cells.
(DOCX)
