# Supplementary material for: Phenotypic, Genomic and Functional Characterization Reveals No Differences between CD138++ and CD138low Subpopulations in Multiple Myeloma Cell Lines
Source: PLoS One. 2014 Mar 21;9(3):e92378. doi: 10.1371/journal.pone.0092378 (PMC3962421; doi:10.1371/journal.pone.0092378)
Supplement: Figure S1 — Gating strategy for sorting CD138++ and CD138low subpopulations in RPMI-8226 cells. Top panel: viable cells were gated as 7AAD-ve (R1) and subsequently debris were eliminated by scatter properties (R2). Finally, the lowest and highest 5% CD138-expressing cells were gated on R2. Bottom panel: CD138++ and CD138low sorted cells had a final purity >95% in the post-sort analysis. (DOCX) [file pone.0092378.s001.docx]

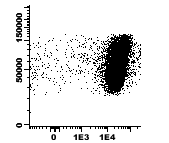

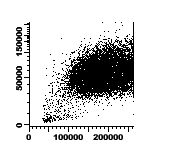


**PRE-SORT**

**POST-SORT**


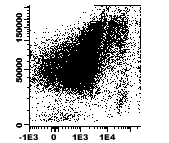


**7AAD**

**SSC**

**FSC**

**SSC**

**CD138**

**SSC**

**5%**

**5%**

**CD138^low^**


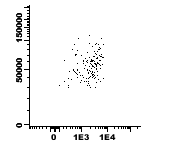


**>95% PURITY**

**CD138^++^**


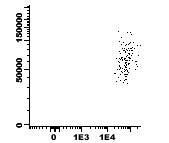


**R1**

**R1**

**R2**

**R2**

**>95% PURITY**

**CD138**

**SSC**

**Figure S1. Gating strategy for sorting CD138^++^ and CD138^low^ subpopulations in RPMI-8226 cells.** Top panel: viable cells were gated as 7AAD^-ve^ (R1) and subsequently debris were eliminated by scatter properties (R2). Finally, the lowest and highest 5% CD138-expressing cells were gated on R2. Bottom panel: CD138^++^ and CD138^low^ sorted cells had a final purity > 95% in the post-sort analysis.
