# Supplementary material for: Phenotypic, Genomic and Functional Characterization Reveals No Differences between CD138++ and CD138low Subpopulations in Multiple Myeloma Cell Lines
Source: PLoS One. 2014 Mar 21;9(3):e92378. doi: 10.1371/journal.pone.0092378 (PMC3962421; doi:10.1371/journal.pone.0092378)
Supplement: Figure S2 — Morphology of CD138++ and CD138low RPMI-8226 cells. May-Grünwald-Giemsa staining of sorted CD138++ and CD138low RPMI-8226 cells. Scale bar = 10 μm. (DOCX) [file pone.0092378.s002.docx]

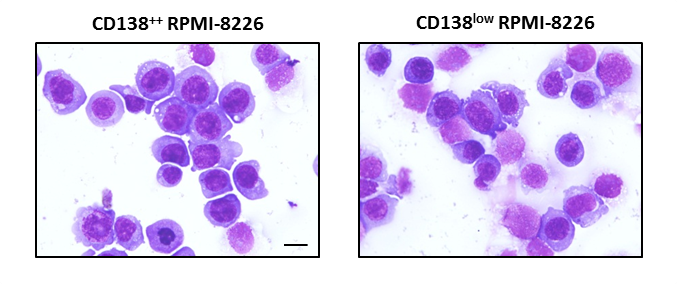


**Figure S2. Morphology of CD138^++^ and CD138^low^ RPMI-8226 cells.** May-Grünwald-Giemsa staining of sorted CD138^++^ and CD138^low^ RPMI-8226 cells. Scale bar = 10 µm.
