# Supplementary material for: Phenotypic, Genomic and Functional Characterization Reveals No Differences between CD138++ and CD138low Subpopulations in Multiple Myeloma Cell Lines
Source: PLoS One. 2014 Mar 21;9(3):e92378. doi: 10.1371/journal.pone.0092378 (PMC3962421; doi:10.1371/journal.pone.0092378)
Supplement: Figure S3 — Analysis of apoptosis in CD138++ and CD138low subpopulations in RPMI-8226, NCI-H929, MM1S and U266 cell lines. Non-apoptotic cells were gated as annexin-V-ve/7AAD-ve (R1) and subsequently debris were eliminated by scatter properties (R2). The third dot plot for every MM cell line corresponding to R2 shows the percentage of CD138low cells. (DOCX) [file pone.0092378.s003.docx]

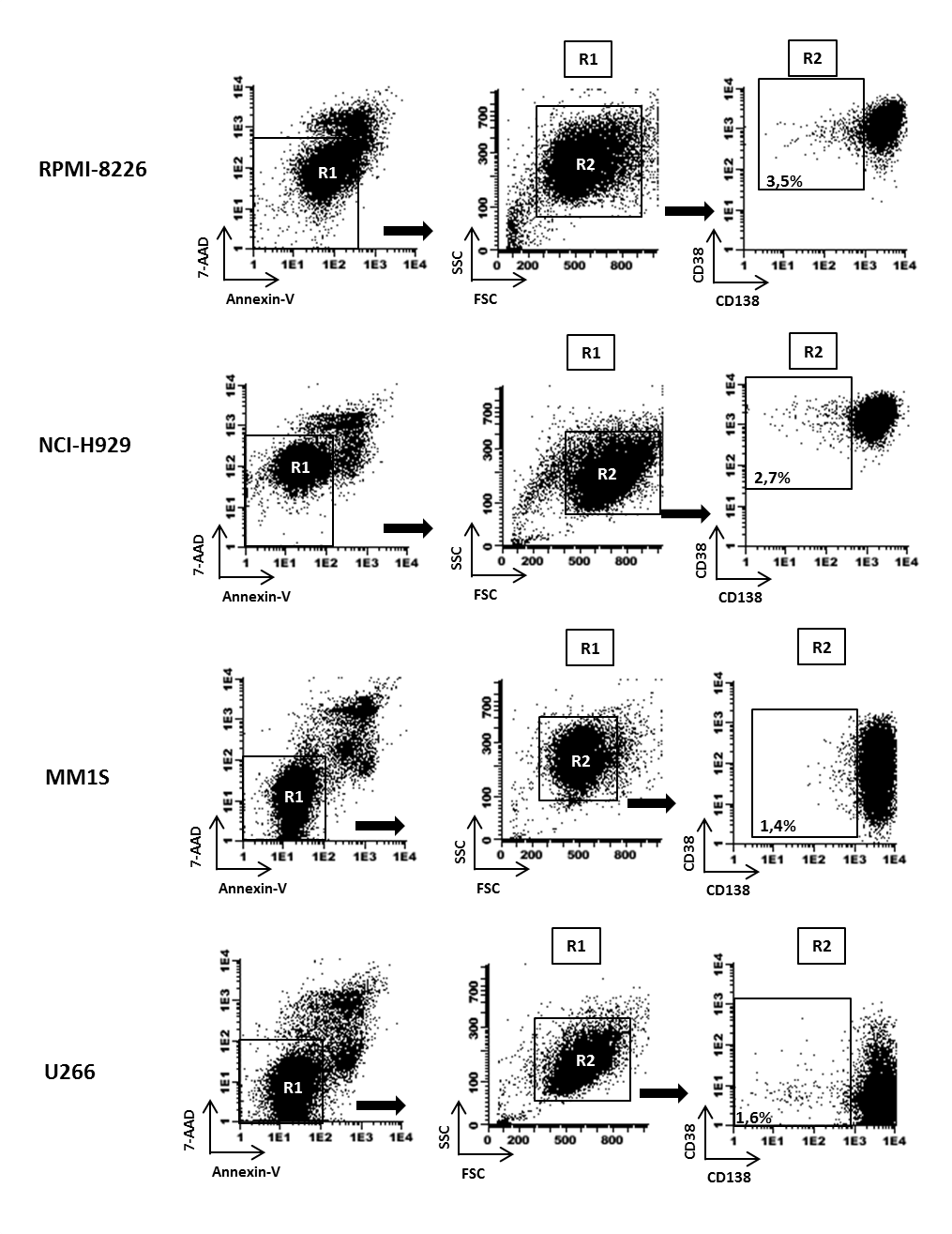


**Figure S3. Analysis of apoptosis in CD138^++^ and CD138^low^ subpopulations in RPMI-8226, NCI-H929, MM1S and U266 cell lines.** Non-apoptotic cells were gated as annexin-V^-ve^/7AAD^-ve^ (R1) and subsequently debris were eliminated by scatter properties (R2). The third dot plot for every MM cell line corresponding to R2 shows the percentage of CD138^low^ cells.
